# Morphological and Molecular Data Reveal Two New Cryptic Spider Species of the *Pholcus yichengicus* Species Group (Araneae, Pholcidae) from Central China †

**DOI:** 10.3390/ani16121884

**Published:** 2026-06-18

**Authors:** Mei-Chen Yan, Zhi-Yuan Yao

**Affiliations:** College of Life Science, Shenyang Normal University, Shenyang 110034, China

**Keywords:** biodiversity, DNA barcode, East Asia, invertebrate, molecular species delimitation, taxonomy

## Abstract

Two new cryptic species from central China, belonging to the *Pholcus yichengicus* species group, were identified using an integrative approach combining morphological and three molecular species delimitation methods, and incorporating data from five related previously described species. These two new species are named *Pholcus chengkou* **sp. nov.** and *P. yanan* **sp. nov.** DNA barcodes were generated for the two new species to calculate p-distances and K2P distances, thus confirming their identities alongside the five previously described species. Additionally, revised diagnoses for the five previously described species and a distribution map encompassing all the species discussed are provided.

## Simple Summary

Twenty-three spider species of *Pholcus* have been documented in the Qinling Mountains of central China. This paper presents the identification of two new cryptic species from the vicinity of the Qinling Mountains, based on morphology and three methods of molecular species delimitation (ABGD, GMYC, and bPTP), incorporating data from five related previously described species. All of which belong to the *Pholcus yichengicus* species group. Additionally, revised diagnoses for the five previously described species are also provided.

## 1. Introduction

The family Pholcidae C.L. Koch, 1850 represents a highly diverse group of spiders, encompassing 97 genera and 2060 species [1]. These species are classified into seven subfamilies: Arteminae Simon, 1893, Caipirinae Meng et al., 2026, Modisiminae Simon, 1893, Ninetinae Simon, 1890, Pholcinae C.L. Koch, 1850, Physocyclinae Meng et al., 2026, and Smeringopinae Simon, 1893 [2,3,4,5]. Within Pholcinae, the genus *Pholcus* Walckenaer, 1805 stands out as the most diverse, with 434 described species grouped into 21 species groups [1,4,6]. These species are predominantly distributed across the Palaearctic, Oriental, Afrotropical, and Australasian biogeographic realms [1,7].

Recently, a series of studies on *Pholcus* have been conducted in China, utilizing both morphological and molecular data. The survey areas encompass the Changbai Mountains, the mountainous regions between the Changbai and Yanshan-Taihang Mountains in northeastern China [8,9,10,11,12,13], the Yanshan-Taihang Mountains and the Lüliang Mountains in northern China [14,15,16], the Qinling Mountains in central China [17,18], and the surrounding areas of the Sichuan Basin in southwestern China [19,20]. To date, China exhibits the highest diversity of *Pholcus* species [1,21], with 195 species recorded, accounting for 45% of the global total for this genus [1].

**Figure 1:**
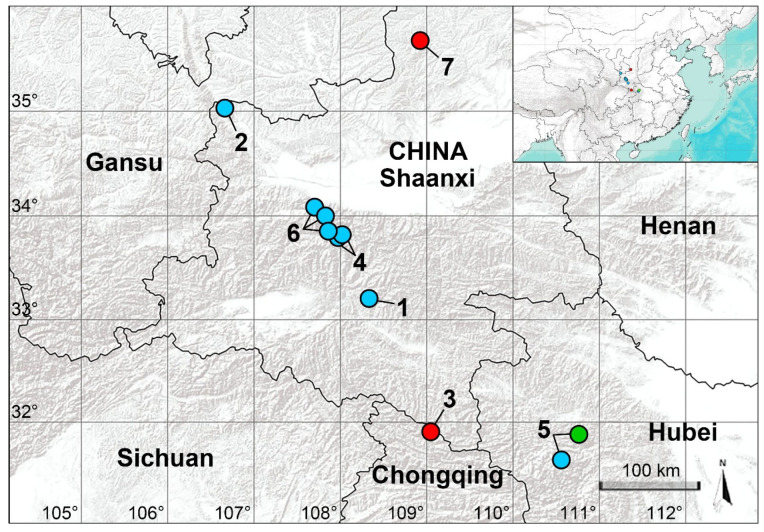
Distribution records of species of the *Pholcus yichengicus* species group from the Qinling Mountains and neighboring area. 1: *P. ankang*; 2: *P. baoji*; 3: *P. chengkou* sp. nov.; 4: *P. ovatus*; 5: *P. songi*; 6: *P. taibaiensis*; 7: *P. yanan* sp. nov. Blue, green, and red circles represent previously recorded species, known species collected in this study, and new species, respectively.

The Qinling Mountains in China are generally recognized as a geographical demarcation line between northern and southern China, spanning the provinces of Shanxi, Shaanxi, and Henan. As of now, 23 species of *Pholcus* have been documented in the Qinling Mountains [17,18]. During our examination of specimens collected from the vicinity of the Qinling Mountains, we identified two species that are morphologically similar to their congeners but genetically distinct, qualifying them as cryptic species. Here, we describe these two new cryptic species based on both morphological and molecular evidence, incorporating data from five related previously described species (Figure 1). All of which belong to the *Pholcus yichengicus* species group.

## 2. Materials and Methods

Specimens were examined and measured with a Leica M205 C stereomicroscope. Left male palps were photographed (unless otherwise indicated in figure legends). Epigynes were photographed before dissection. Vulvae were photographed after treatment in a warm 10% solution of potassium hydroxide (KOH) to dissolve soft tissues. Images were captured with a Canon EOS 750D wide zoom digital camera (24.2 megapixels, Tokyo, Japan) mounted on the stereomicroscope mentioned above and assembled using Helicon Focus v3.10.3 image stacking software [22]. All measurements are given in millimeters (mm). Leg measurements are shown as: total length (femur, patella, tibia, metatarsus, tarsus). Leg segments were measured on their dorsal side. The distribution map was generated with ArcGIS v. 10.2 (ESRI Inc., Redlands, CA, USA). The specimens studied are preserved in 75% ethanol and are deposited in the College of Life Science, Shenyang Normal University (SYNU) in Shenyang, Liaoning, China, the Institute of Zoology, Chinese Academy of Sciences (IZCAS) in Beijing, China, and the Museum of Hebei University (MHBU) in Baoding, Hebei, China.

Terminology and taxonomic descriptions follow Huber [6] and Yao et al. [9,23]. The following abbreviations are used: **a** = appendix, **aa** = anterior arch, **ALE** = anterior lateral eye, **AME** = anterior median eye, **b** = bulb, **da** = distal apophysis, **dp** = distal process, **ds** = dorsal spine, **e** = embolus, **fa** = frontal apophysis, **kn** = knob, **L/d** = length/diameter ratio, **mb** = median branch, **pa** = proximo-lateral apophysis, **PME** = posterior median eye, **pp** = pore plate, **pr** = procursus, **pra** = proximal apophysis, **psa** = prolatero-subdistal apophysis, **pvp** = prolatero-ventral protrusion, **rdp** = retrolatero-distal process, **rpa** = retrolatero-proximal apophysis, **u** = uncus, **va** = ventral apophysis, **vp** = ventral protrusion.

The mitochondrial gene fragments encoding *COI* were obtained for 11 samples (Table 1), using the following primers: forward: *COI*Jerry 2 (5’-CAGCATTTGTTTTGATTTTTTGG-3’) and reverse: C1-N-2776 (5’-GGATAATCAGAATATCGTCGAGG-3’) [24]. Two species, *Pholcus paralinzhou* Zhang & Zhu, 2009 and *P. taishan* Song & Zhu, 1999, were selected as outgroups. DNA sequences were checked and edited with BioEdit 7.2.2 [25]. P-distances and K2P distances from *COI* were computed using MEGA 5 [26]. Phylogenetic trees were constructed using the Maximum Likelihood (ML) method for molecular species delimitation. ML analyses were conducted using RAxML 8.2.9 under a GTRCAT model for all partitions, with 500 rapid bootstrap replicates followed by a thorough Maximum Likelihood tree search [27]. The sequences are deposited in GenBank. For additional information on extraction, amplification and sequencing procedures, see Yao et al. [28] and Yao and Li [7].

We applied three methods for molecular species delimitation. The Automatic Barcode Gap Discovery (ABGD) analyses were conducted using both Jukes–Cantor and Kimura 2-P distance matrices with options: Pmin = 0.001, Pmax = 0.1, Steps = 10, X = 1.0, Nb bins = 20 [29]. The Bayesian implementation of the Poisson Tree Processes (bPTP) analysis was run for 100,000 generations, with a thinning of 100 and burn-in of 0.25 [30]. The Generalised Mixed Yule Coalescent (GMYC) analysis was performed under the single threshold model using the R 4.5.2 package SPLITS [31]. The phylogenetic tree was converted to an ultrametric format for GMYC analysis using BEAST 1.8.2 [32].

## 3. Results

**Figure 2:**
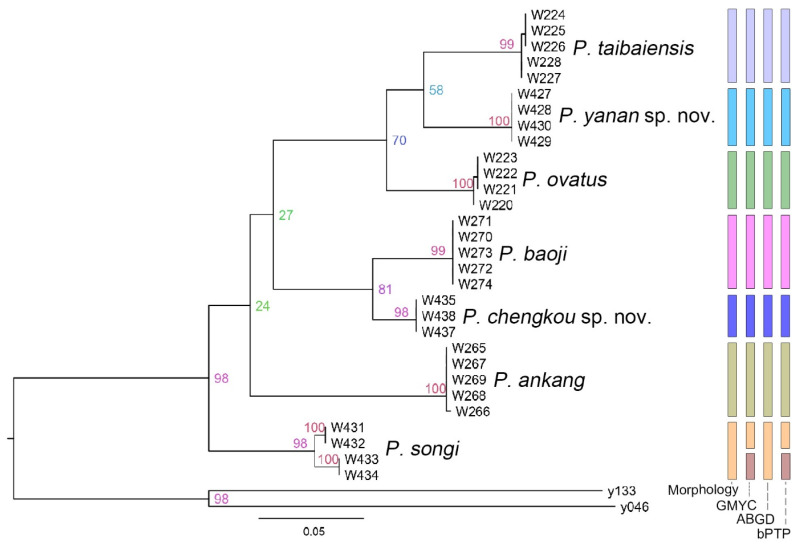
Results of the species delimitation analysis conducted using morphology (visual examination of characters), GMYC, ABGD and bPTP; different colors of the bars represent the different species. The phylogenetic tree was inferred from ML analysis, bootstrap values are provided at the nodes, and branch lengths are scaled to the number of substitutions per site.

We obtained an alignment of 566 bp of the *COI* gene. Figure 2 shows the phylogenetic tree constructed based on the *COI* data. The tree clearly divided the samples into seven deeply divergent clades. The ABGD analysis identified seven provisional species using both the Jukes–Cantor and Kimura 2-P distance matrices, and the results were fairly consistent with morphological evidence (the visual examination of characters). The GMYC and bPTP analyses, however, identified eight species, with *Pholcus songi* Zhang & Zhu, 2009 (as identified through morphology and ABGD) being split into two species (Figure 2: light brown and brown boxes).

The average genetic distances among the seven major clades and the maximum distances within them, estimated from the *COI* data, are presented in Table 2. Both uncorrected p-distances and the K2P distances were estimated. The p-distances and K2P distances ranged from 0.044 to 0.109 and from 0.046 to 0.119, respectively, among the major clades. Within these clades, the p-distances ranged from 0 to 0.009.

## 4. Discussion

We classified the seven major clades as seven candidate species because the ABGD analysis provides support for speciation events among the seven clades, and the results are fairly consistent with the morphological evidence. Although the GMYC and bPTP analyses split the species *P. songi* into two species, these two delimitation outcomes are unreasonable. This is because all four samples (W431–W434) originate from the same population. Moreover, the maximum p-distance among them is mere 0.009. Additionally, the morphological characters of specimens from the same population are also consistent, e.g., the raised prolatero-subdistal membranous edge of the procursus is laterally pointed (figure 157D in Yao and Li [33]), the appendix is distally pointed (figure 157A in Yao and Li [33]), the median branch of the appendix as long as the appendix (figure 157A in Yao and Li [33]), and the vulval pore plates are close to the posterior part of the anterior arch (figure 158B in Yao and Li [33]). More detailed diagnoses, descriptions, and photomicroscopy images of new species as well as revised diagnoses for the five previously described species are provided under the Taxonomy section (see below).

## 5. Taxonomy

Family Pholcidae C.L. Koch, 1850

Subfamily Pholcinae C.L. Koch, 1850

Genus *Pholcus* Walckenaer, 1805

Type species. *Aranea phalangioides* Fuesslin, 1775 from Switzerland.

### Pholcus yichengicus Species Group

Remarks. This species group can be distinguished by the following combination of characters: male chelicerae with proximo-lateral, distal, and frontal apophyses (pa, da, fa); male palpal trochanter apophyses retrolatero-proximally strongly bulged; male palpal tibia with prolatero-ventral protrusion (pvp); procursus (pr) with dorsal spines (ds); appendix (a) usually branched; epigyne sclerotized, with knob (kn) [6,17,34]. The new species described below are assigned to this group because their morphological characters are consistent with those listed above. This species group comprises 52 previously described species and is primarily distributed in central and southern China, as well as northern Thailand [6,11,15,17,18,19,34]. Among these, 48 species have been recorded from China. Two new species are described below.

***Pholcus chengkou* sp. nov.**

LSID: urn:lsid:zoobank.org:act:ADAB463F-4FEE-41CA-904F-DE4AC5C4BF5A

**Figure 3:**
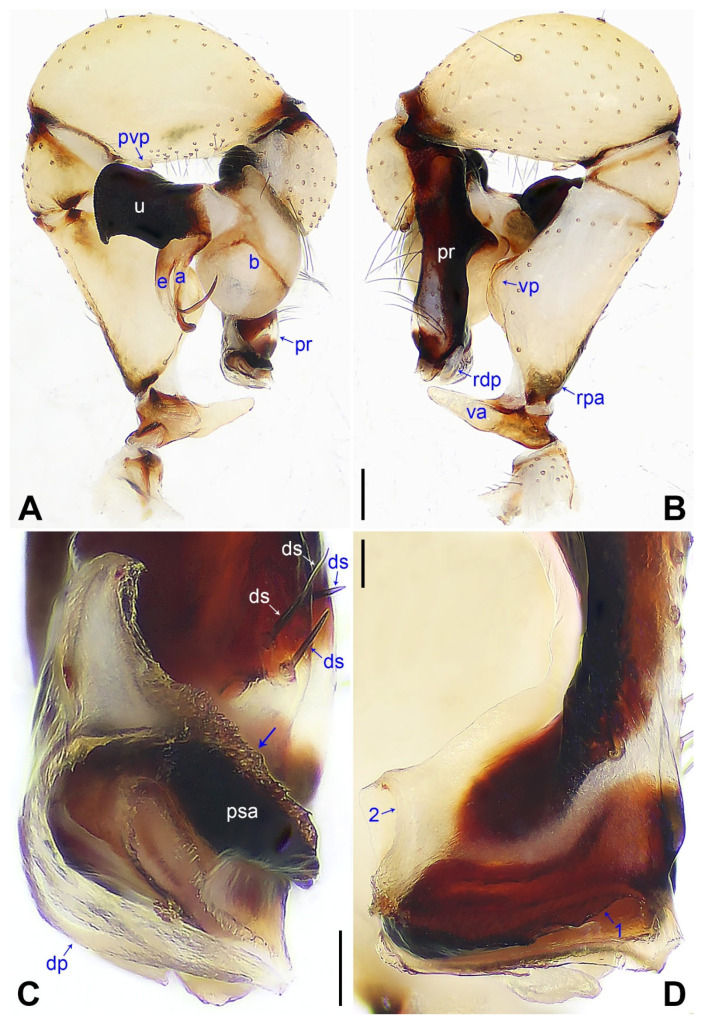
*Pholcus chengkou* sp. nov., holotype male: (**A**) palp, prolateral view; (**B**) palp, retrolateral view; (**C**) distal part of procursus, prolateral view, arrow indicates membranous edge; (**D**) distal part of procursus, dorsal view, arrow 1 indicates latero-distal part of prolatero-subdistal apophysis of procursus, arrow 2 indicates lateral part of raised prolatero-subdistal membranous edge of procursus. Abbreviations: a = appendix, b = bulb, dp = distal process, ds = dorsal spine, e = embolus, pr = procursus, psa = prolatero-subdistal apophysis, pvp = prolatero-ventral protrusion, rdp = retrolatero-distal process, rpa = retrolatero-proximal apophysis, u = uncus, va = ventral apophysis, vp = ventral protrusion. Scale bars: (**A**,**B**) = 0.20 mm; (**C**,**D**) = 0.05 mm.

**Figure 4:**
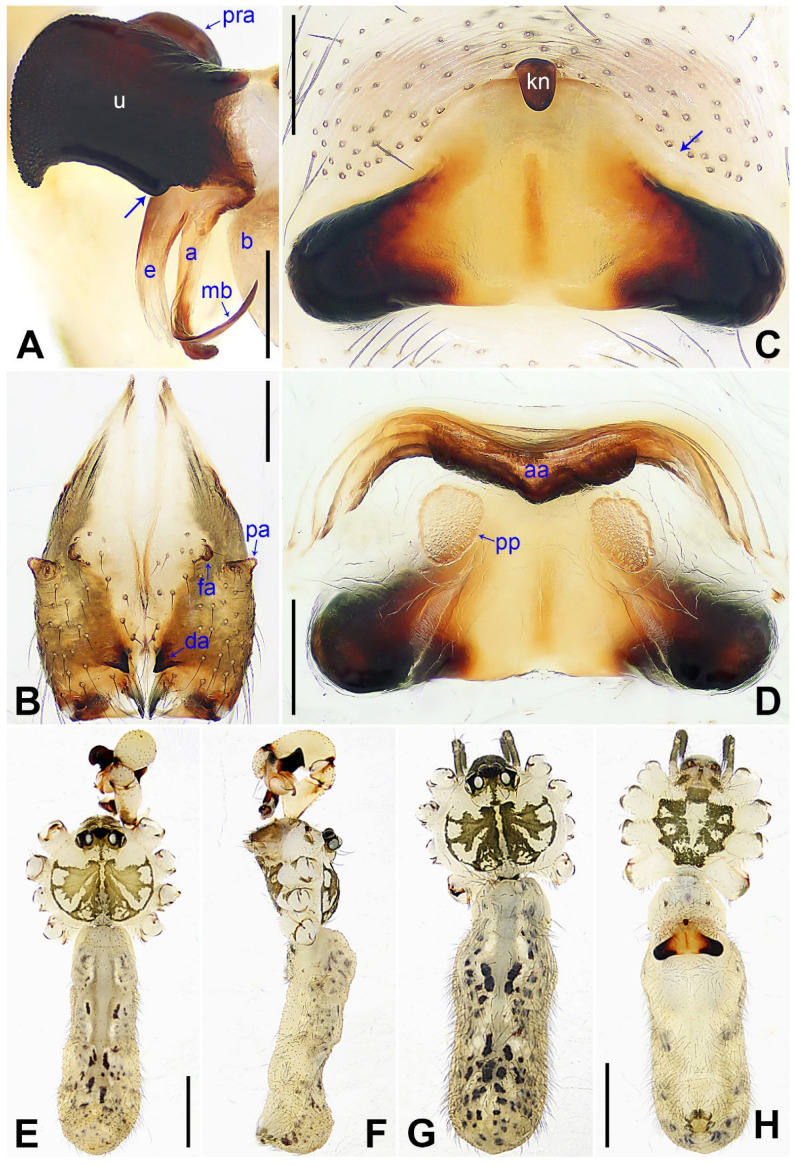
*Pholcus chengkou* sp. nov., holotype male (**A**,**B**,**E**,**F**) and paratype female (**C**,**D**,**G**,**H**): (**A**) bulbal apophyses, prolateral view, arrow indicates slightly protruding latero-subproximal part of uncus; (**B**) chelicerae, frontal view; (**C**) epigyne, ventral view, arrow indicates contracted antero-lateral part; (**D**) vulva, dorsal view; (**E**), (**G**) habitus, dorsal view; (**F**) habitus, lateral view; (**H**) habitus, ventral view. Abbreviations: a = appendix, aa = anterior arch, b = bulb, da = distal apophysis, e = embolus, fa = frontal apophysis, kn = knob, mb = median branch, pa = proximo-lateral apophysis, pp = pore plate, pra = proximal apophysis, u = uncus. Scale bars: (**A**–**D**) = 0.20 mm; (**E**–**H**) = 1.00 mm.

Figure 3 and Figure 4

**Type material.** ***Holotype*:** CHINA • ♂; Chongqing, Chengkou County, Heyu Town, Liangshanmen Village, roadside of Y004; 31.905585° N, 109.048853° E; alt. 1563 m; 5 August 2025; Zhi-Yuan Yao, Bing Wang & Xin-Yu Li leg.; SYNU-Ar00534. ***Paratypes*:** CHINA • 6♀; same data as for the holotype; SYNU-Ar00535–540.

**Etymology.** The specific name refers to the type locality; noun in apposition.

**Diagnosis.** The new species resembles *Pholcus baoji* Yang, He & Yao, 2024 (Yang et al. 2024: 282, figures 5A–D and 6A–H [18]) by having similar male chelicerae (Figure 4B), but it can be distinguished by the sclerotized prolatero-subdistal apophysis of the procursus not protruding latero-distally (arrow 1 in Figure 3D vs. strongly protruding) and with a membranous edge (arrow in Figure 3C vs. absent), by the raised prolatero-subdistal membranous edge of the procursus laterally curved (arrow 2 in Figure 3D vs. laterally rectangular), by the uncus slightly protruding latero-subproximally (arrow in Figure 4A vs. strongly protruding latero-medially), by the epigyne contracted antero-laterally (arrow in Figure 4C vs. not contracted), and by the vulval anterior arch strongly curved medially (M-shaped, aa in Figure 4D vs. straight medially).

**Description. Male** (***holotype***): Measurements: Total length 4.66 (4.81 with clypeus), carapace 1.44 long, 1.76 wide, opisthosoma 3.22 long, 0.95 wide. Leg I: missing, leg II: 28.67 (8.01, 0.69, 7.15, 11.28, 1.54), leg III: 19.05 (5.48, 0.62, 4.75, 7.05, 1.15), leg IV: 26.08 (7.56, 0.64, 6.47, 10.00, 1.41). Eye interdistances and diameters: PME–PME 0.23, PME 0.16, PME–ALE 0.06, AME–AME 0.06, AME 0.14. Sternum width/length: 1.06/0.99.

Color: Carapace yellowish, with brown radiating marks and marginal brown bands; ocular area yellowish, with anterior brown marks and median brown band; clypeus and sternum yellowish, with brown marks. Legs yellowish, but brown on patellae and distal parts of femora and tibiae, with darker rings on submedian and subdistal parts of femora and subproximal, submedian, and subdistal parts of tibiae. Opisthosoma yellowish, with dorsal and lateral brown spots.

Body: As in Figure 4E,F; ocular area elevated, without eye-stalks.

Chelicerae: As in Figure 4B, with pair of proximo-lateral apophyses (pa), pair of distal apophyses (da) with two teeth each, and pair of frontal apophyses (fa).

Palp: As in Figure 3A,B; trochanter with long (4× longer than wide), strongly bulged ventral apophysis (va) retrolaterally; femur with retrolatero-proximal apophysis (rpa) and distinct ventral protrusion (vp); procursus (pr) simple proximally, but complex distally, with raised prolatero-subdistal membranous edge bearing distal membranous process (dp), sclerotized prolatero-subdistal apophysis (psa) with membranous edge (arrow in Figure 3C), retrolatero-distal membranous process (rdp), and three strong and one slender dorsal spines (ds); uncus (u) protruding latero-subproximally (arrow in Figure 4A), with proximal apophysis (pra) and distal scaly edge; appendix (a) hooked and blunt distally, with median branch (mb; as long as appendix); embolus (e) weakly sclerotized, with some transparent distal projections.

Legs: Legs with short vertical setae on tibiae, metatarsi, and tarsi.

**Female** (***paratype***, SYNU-Ar00535): Similar to male, habitus as in Figure 4G,H. Total length 4.62 (4.79 with clypeus), carapace 1.34 long, 1.60 wide, opisthosoma 3.28 long, 1.11 wide; tibia I: 7.44; tibia I L/d: 50. Eye interdistances and diameters: PME–PME 0.22, PME 0.15, PME–ALE 0.04, AME–AME 0.03, AME 0.14. Sternum width/length: 1.01/0.98. Epigyne (Figure 4C) nearly triangular, but contracted antero-laterally (arrow in Figure 4C), strongly sclerotized laterally, with column-shaped knob (kn). Vulva (Figure 4D) with strongly curved (M-shaped) anterior arch (aa) laterally and medially and pair of nearly elliptic pore plates (pp).

**Variation.** Tibia I in the other five female paratypes (SYNU-Ar00536–540): 7.37–7.56.

**Habitat.** Underside of overhang on rocky cliffs in a mountainous area.

**Distribution.** China (Chongqing, known only from the type locality; Figure 1).

***Pholcus yanan* sp. nov.**

LSID: urn:lsid:zoobank.org:act:D01686BC-1DC8-4B07-91C9-DB236D2B98F9

**Figure 5:**
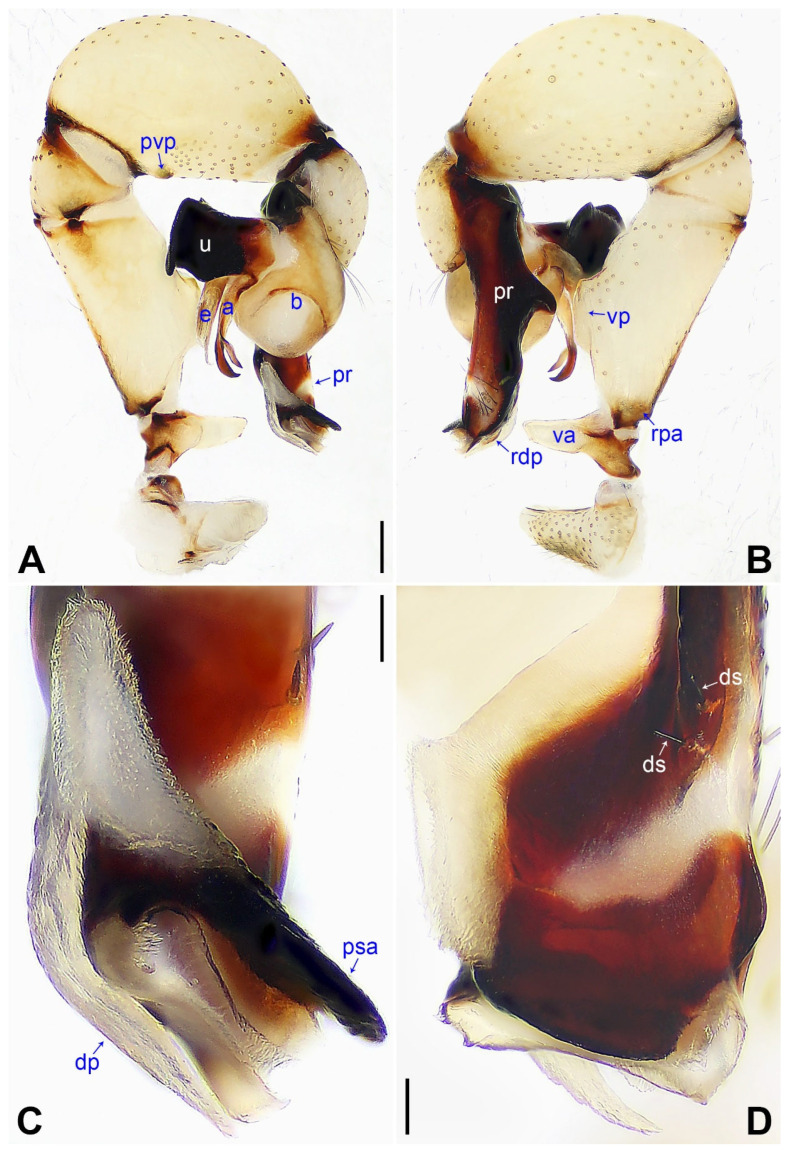
*Pholcus yanan* sp. nov., holotype male: (**A**) flipped right palp, prolateral view; (**B**) flipped right palp, retrolateral view; (**C**) distal part of flipped right procursus, prolateral view; (**D**) distal part of flipped right procursus, dorsal view. Abbreviations: a = appendix, b = bulb, dp = distal process, ds = dorsal spine, e = embolus, pr = procursus, psa = prolatero-subdistal apophysis, pvp = prolatero-ventral protrusion, rdp = retrolatero-distal process, rpa = retrolatero-proximal apophysis, u = uncus, va = ventral apophysis, vp = ventral protrusion. Scale bars: (**A**,**B**) = 0.20 mm; (**C**,**D**) = 0.05 mm.

**Figure 6:**
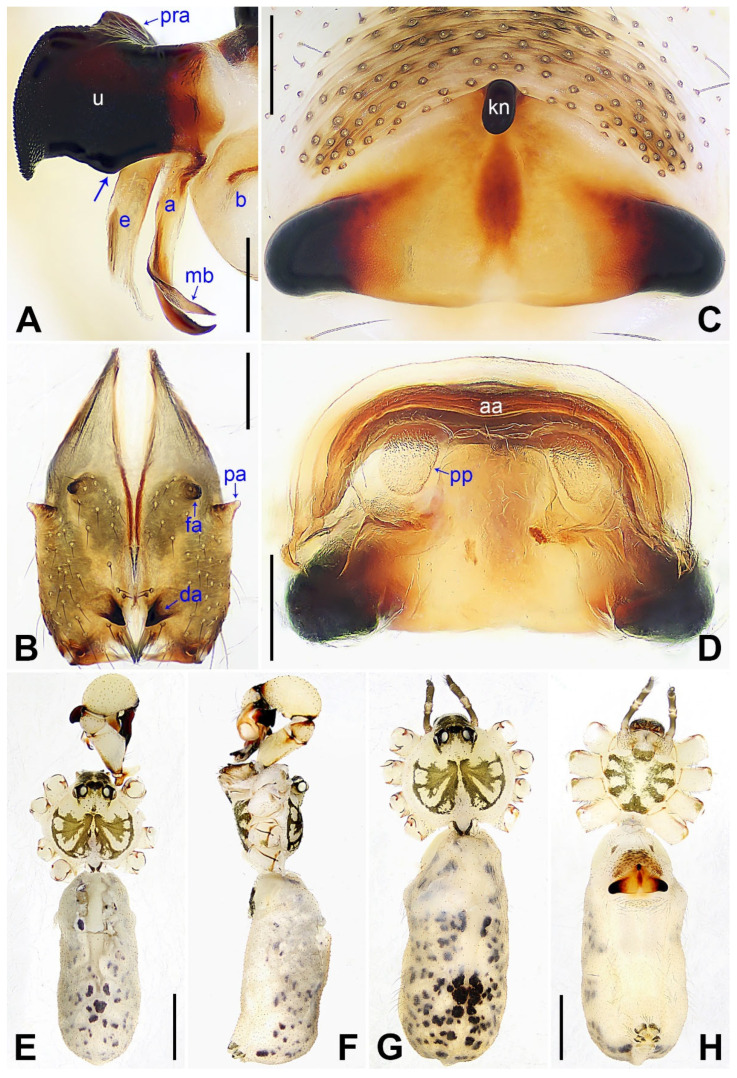
*Pholcus yanan* sp. nov., holotype male (**A**,**B**,**E**,**F**) and paratype female (**C**,**D**,**G**,**H**): (**A**) flipped right bulbal apophyses, prolateral view, arrow indicates protruding latero-median part of uncus; (**B**) chelicerae, frontal view; (**C**) epigyne, ventral view; (**D**) vulva, dorsal view; (**E**), (**G**) habitus, dorsal view; (**F**) habitus, lateral view; (**H**) habitus, ventral view. Abbreviations: a = appendix, aa = anterior arch, b = bulb, da = distal apophysis, e = embolus, fa = frontal apophysis, kn = knob, mb = median branch, pa = proximo-lateral apophysis, pp = pore plate, pra = proximal apophysis, u = uncus. Scale bars: (**A**–**D**) = 0.20 mm; (**E**–**H**) = 1.00 mm.

Figure 5 and Figure 6

**Type material.** ***Holotype*:** CHINA • ♂; Shaanxi, Yan’an, Huangling County, Shuanglong Town, Wan’an Temple Scenic Spot; 35.669395° N, 108.931323° E; alt. 1032 m; 28 July 2025; Zhi-Yuan Yao, Bing Wang & Xin-Yu Li leg.; SYNU-Ar00550. ***Paratypes*:** CHINA • 1♂; same data as for the holotype; SYNU-Ar00551 • 7♀; same data as for the holotype; SYNU-Ar00552–558.

**Etymology.** The specific name refers to the type locality; noun in apposition.

**Diagnosis.** The new species resembles *Pholcus taibaiensis* Wang & Zhu, 1992 (Yao and Li 2012: 34, figures 169A–D and 170A–C [33]) by having similar male chelicerae and uncus (Figure 6A,B), but it can be distinguished by the sclerotized prolatero-subdistal apophysis of the procursus elongated distally in prolateral view (psa in Figure 5C vs. not elongated), by the appendix sclerotized distally and pointed (a in Figure 6A vs. not sclerotized distally, blunt), by the median branch of the appendix 0.5× longer than the appendix (mb in Figure 6A vs. 0.25×), and by the epigynal plate nearly straight posteriorly (Figure 6C,D vs. curved).

**Description. Male** (***holotype***): Measurements: Total length 4.48 (4.60 with clypeus), carapace 1.25 long, 1.52 wide, opisthosoma 3.23 long, 1.28 wide. Leg I: 35.59 (9.01, 0.61, 9.10, 14.74, 2.13), leg II: 25.03 (6.86, 0.59, 6.28, 10.00, 1.30), leg III: 17.71 (5.13, 0.56, 4.42, 6.57, 1.03), leg IV: 22.53 (6.44, 0.57, 5.58, 8.65, 1.29); tibia I L/d: 65. Eye interdistances and diameters: PME–PME 0.25, PME 0.15, PME–ALE 0.04, AME–AME 0.05, AME 0.11. Sternum width/length: 1.00/0.88.

Color: Carapace yellowish, with brown radiating marks and marginal brown bands; ocular area yellowish, with anterior brown marks; clypeus and sternum yellowish, with brown marks. Legs yellowish, but dark brown on patellae and whitish on distal parts of femora and tibiae, with darker rings on submedian and subdistal parts of femora and proximal and subdistal parts of tibiae. Opisthosoma yellowish, with dorsal and lateral brown spots.

Body: As in Figure 6E,F; ocular area elevated, without eye-stalks.

Chelicerae: As in Figure 6B, with pair of proximo-lateral apophyses (pa), pair of distal apophyses (da) with two teeth each, and pair of frontal apophyses (fa).

Palp: As in Figure 5A,B; trochanter with long (2× longer than wide), strongly bulged ventral apophysis (va) retrolaterally; femur with retrolatero-proximal apophysis (rpa) and distinct ventral protrusion (vp); procursus (pr) simple proximally, but complex distally, with raised prolatero-subdistal membranous edge bearing distal membranous process (dp), sclerotized prolatero-subdistal apophysis (psa), retrolatero-distal membranous process (rdp), and two strong dorsal spines (ds); uncus (u) protruding latero-medially (arrow in Figure 6A), with proximal apophysis (pra) and distal scaly edge; appendix (a) curved, hooked distally and pointed, with median branch (mb; 0.5× longer than appendix); embolus (e) weakly sclerotized, with some transparent distal projections.

Legs: Retrolateral trichobothrium on tibia I situated at 3% proximally; legs with short vertical setae on tibiae, metatarsi, and tarsi; tarsus I with 26 distinct pseudosegments.

**Female** (***paratype***, SYNU-Ar00552): Similar to male, habitus as in Figure 6G,H. Total length 5.16 (5.33 with clypeus), carapace 1.48 long, 1.68 wide, opisthosoma 3.68 long, 1.66 wide; tibia I: 9.36; tibia I L/d: 62. Eye interdistances and diameters: PME–PME 0.22, PME 0.16, PME–ALE 0.06, AME–AME 0.04, AME 0.11. Sternum width/length: 1.13/1.00. Epigyne (Figure 6C) nearly triangular, strongly sclerotized laterally and medially, with column-shaped knob (kn). Vulva (Figure 6D) with strongly curved anterior arch (aa) laterally and pair of nearly elliptic pore plates (pp).

**Variation.** Tibia I in male paratype (SYNU-Ar00551): 12.31. Tibia I in the other six female paratypes (SYNU-Ar00553–558): 8.30–8.97.

**Habitat.** Underside of overhang on rocky cliffs in a mountainous area.

**Distribution.** China (Shaanxi, known only from the type locality; Figure 1).

***Pholcus ankang* Yang, He & Yao, 2024**

*Pholcus ankang* Yang et al. 2024: 282, figures 3A–D, 4A–H [18].

**Type material examined.** ***Holotype*:** CHINA • ♂; Shaanxi, Ankang, Shiquan County, Chengguan Town, Guiguling Scenic Spot; 33.205333° N, 108.335833° E; alt. 1585 m; 24 July 2022; Zhi-Yuan Yao, Lan Yang & Lu-Dan Zhang leg.; SYNU-Ar00398. ***Paratypes*:** CHINA • 2♂; same data as for the holotype; SYNU-Ar00399–400 • 2♀; same data as for the holotype; SYNU-Ar00401–402.

**Diagnosis.** The species resembles *Pholcus chengkou* sp. nov. (Figure 3 and Figure 4) by having similar male chelicerae and epigyne (figure 4A,D in Yang et al. [18]), but it can be distinguished by the sclerotized prolatero-subdistal apophysis of the procursus without a membranous edge (arrow 2 in figure 3C in Yang et al. [18] vs. present), by the raised prolatero-subdistal membranous edge of the procursus angular laterally (arrow 3 in figure 3D in Yang et al. [18] vs. curved), by the uncus strongly protruding latero-subproximally (arrow 1 in figure 4C in Yang et al. [18] vs. slightly protruding), by the appendix with the angular median branch (arrow 2 in figure 4C in Yang et al. [18] vs. slender, as long as the appendix), and by the vulval anterior arch straight medially (figure 4B in Yang et al. [18] vs. strongly curved medially, M-shaped).

**Habitat.** Underside of overhang on rocky cliffs in a mountainous area.

**Distribution.** China (Shaanxi; Figure 1).

***Pholcus baoji* Yang, He & Yao, 2024**

*Pholcus baoji* Yang et al. 2024: 285, figures 5A–D and 6A–H [18].

**Type material examined.** ***Holotype*:** CHINA • ♂; Shaanxi, Baoji, Long County, Xinjichuan Town, Longmendong Scenic Spot; 35.038833° N, 106.670333° E; alt. 1489 m; 29 July 2022; Zhi-Yuan Yao, Lan Yang & Lu-Dan Zhang leg.; SYNU-Ar00403. ***Paratypes*:** CHINA • 1♂; same data as for the holotype; SYNU-Ar00404 • 2♀; same data as for the holotype; SYNU-Ar00405–406.

**Diagnosis.** The species resembles *Pholcus chengkou* sp. nov. (Figure 3 and Figure 4) by having similar male chelicerae (figure 6D in Yang et al. [18]), but it can be distinguished by the sclerotized prolatero-subdistal apophysis of the procursus strongly protruding latero-distally (arrow 4 in figure 5D in Yang et al. [18] vs. not protruding) and without a membranous edge (figure 5C in Yang et al. [18] vs. present), by the raised prolatero-subdistal membranous edge of the procursus rectangular laterally (arrow 3 in figure 5D in Yang et al. [18] vs. laterally curved), by the uncus strongly protruding latero-medially (arrow 1 in figure 6C in Yang et al. [18] vs. slightly protruding latero-subproximally), by the epigyne not contracted antero-laterally (figure 6A in Yang et al. [18] vs. contracted), and by the vulval anterior arch straight medially (figure 6B in Yang et al. [18] vs. strongly curved medially, M-shaped).

**Habitat.** Underside of overhang on rocky cliffs in a mountainous area.

**Distribution.** China (Shaanxi; Figure 1).

***Pholcus ovatus* Yao & Li, 2012**

*Pholcus ovatus* Yao and Li 2012: 28, figures 134A–D, 135A–E, 136A–D and 137A–D [33]. Yang et al. 2024: 288 [18].

**Type material examined.** ***Holotype*:** CHINA • ♂; Shaanxi, Xi’an, Zhouzhi County, Banfangzi Town; 33.683333–33.820000° N, 107.650000–108.316667° E; 15 May 1991; Xin-Ping Wang leg.; IZCAS. ***Paratypes*:** CHINA • 2♂; same data as for the holotype; IZCAS • 4♀; same data as for the holotype; IZCAS.

**Other material examined.** CHINA • 1♂; Shaanxi, Xi’an, Zhouzhi County, Banfangzi Town (type locality), roadside of G108; 33.800333° N, 107.984667° E; alt. 1165 m; 31 July 2022; Zhi-Yuan Yao, Lan Yang & Lu-Dan Zhang leg.; SYNU-Ar00120F • 1♀; same data as for preceding; SYNU-Ar00121F.

**Diagnosis.** The species resembles *Pholcus yanan* sp. nov. (Figure 5 and Figure 6) by having similar male chelicerae and uncus (figures 134A and 136A–C in Yao and Li [33]), but it can be distinguished by the sclerotized prolatero-subdistal apophysis of the procursus not protruding latero-distally (figures 134D and 137B in Yao and Li [33] vs. strongly protruding) and not elongated distally in prolateral view (figures 134C and 137A in Yao and Li [33] vs. elongated distally), by the appendix blunt distally (figures 134A and 136A in Yao and Li [33] vs. pointed distally), and by the vulval anterior arch strongly protruding postero-medially (figures 135B and 137D in Yao and Li [33] vs. not protruding)

**Habitat.** Underside of overhang on rocky cliffs in a mountainous area.

**Distribution.** China (Shaanxi; Figure 1).

***Pholcus songi* Zhang & Zhu, 2009**

*Pholcus songi* Zhang and Zhu 2009: 80, figure 45A–I [35]. Yao and Li 2012: 32, figures 157A–D and 158A–C [33].

**Type material examined.** ***Holotype*:** CHINA • ♂; Hubei, Shiyan, Fang County, Shennongjia Mountain; 31.700000° N, 110.600000° E; 22 September 2001; Ming-Sheng Zhu, Zi-Zhong Yang leg.; MHBU. ***Paratype*:** CHINA • 1♀; same data as for the holotype; MHBU.

**Other material examined.** CHINA • 2♂; Hubei, Shiyan, Fang County, Yerengu Town, Qinghaoping Village, roadside of G209; 31.886989° N, 110.771395° E; alt. 1344 m; 31 July 2025; Zhi-Yuan Yao, Bing Wang & Xin-Yu Li leg.; SYNU-Ar00541–542 • 7♀; same data as for preceding; SYNU-Ar00543–549.

**Diagnosis.** The species resembles *Pholcus baoji* Yang, He & Yao, 2024 (Yang et al. 2024: 285, figures 5A–D and 6A–H [18]) by having similar male chelicerae, uncus, and epigyne (figures 157A and 158A in Yao and Li [33]), but it can be distinguished by the raised prolatero-subdistal membranous edge of the procursus pointed laterally (figure 157D in Yao and Li [33] vs. rectangular laterally), by the appendix pointed distally (figure 157A in Yao and Li [33] vs. blunt), and by the vulval pore plates close to the posterior part of the anterior arch (figure 158B in Yao and Li [33] vs. separated).

**Habitat.** Underside of overhang on rocky cliffs in a mountainous area.

**Distribution.** China (Hubei; Figure 1).

***Pholcus taibaiensis* Wang & Zhu, 1992**

*Pholcus taibaiensis* Wang and Zhu 1992: 20, figures 1–6 [36]. Song et al. 1999: 63, figure 25I–K [37]. Zhang and Zhu 2009: 90, figure 52A–I [35]. Huber 2011: 451, figures 2097–2099, 2124, 2125, 2178–2183, 2185, 2199 [6]. Yao and Li 2012: 34, figures 169A–D and 170A–C [33]. Yang et al. 2024: 288 [18].

**Type material examined.** ***Holotype*:** CHINA • ♂; Shaanxi, Mei County, Taibai Mountain, Haopingsi Temple; 34.000000° N, 107.833333° E; 6–10 August 1989; Xin-Ping Wang leg.; IZCAS. **‘**Allotype’**:** CHINA • ♀; same data as for the holotype; IZCAS. ***Paratypes*:** CHINA • 1♂; same data as for the holotype; MHBU •1♀; same data as for the holotype; MHBU.

**Other material examined.** CHINA • 3♂; Shaanxi, Baoji, Mei County, Yingtou Town, Haopingsi Temple (type locality); 34.086667° N, 107.705500° E; alt. 1101 m; 30 July 2022; Zhi-Yuan Yao, Lan Yang & Lu-Dan Zhang leg.; SYNU-Ar00130F–132F • 3♀; same data as for preceding; SYNU-Ar00133F–135F.

**Diagnosis.** The species resembles *Pholcus yanan* sp. nov. (Figure 5 and Figure 6) by having similar male chelicerae and uncus (figure 169A in Yao and Li [33]), but it can be distinguished by the sclerotized prolatero-subdistal apophysis of the procursus not elongated distally in prolateral view (figure 169C in Yao and Li [33] vs. elongated distally), by the appendix not sclerotized distally and blunt (figure 169A in Yao and Li [33] vs. sclerotized distally and pointed), by the median branch of the appendix 0.25× longer than the appendix (figure 169A in Yao and Li [33] vs. 0.5×), and by the epigynal plate curved posteriorly (figure 170A in Yao and Li [33] vs. nearly straight).

**Habitat.** Underside of overhang on rocky cliffs in a mountainous area.

**Distribution.** China (Shaanxi; Figure 1).

## 6. Conclusions

Based on morphological and molecular data and comparisons with five previously described closely related species, this study identified and described two new cryptic spider species. This finding enriches our understanding of the cryptic species diversity of this spider group in the Qinling Mountains and surrounding regions.

## Figures and Tables

**Table 1:** Voucher specimen information.

Species	Voucher Code	GenBank Accession Number	Collection Locality	Source
*P. ankang*	W265	PP082941	China, Shaanxi, Ankang	Yang et al. [18]
	W266	PP082942		
	W267	PP082943		
	W268	PP082944		
	W269	PP082945		
*P. baoji*	W270	PP082946	China, Shaanxi, Baoji	Yang et al. [18]
	W271	PP082947		
	W272	PP082948		
	W273	PP082949		
	W274	PP082950		
*P. chengkou* sp. nov.	W435 (SYNU-Ar00538)	PZ470738	China, Chongqing, Chengkou	This study
	W437 (SYNU-Ar00539)	PZ470739		
	W438 (SYNU-Ar00540)	PZ470740		
*P. ovatus*	W220	PP082951	China, Shaanxi, Xi’an	Yang et al. [18]
	W221	PP082952		
	W222	PP082953		
	W223	PP082954		
*P. songi*	W431 (SYNU-Ar00546)	PZ470734	China, Hubei, Shiyan	This study
	W432 (SYNU-Ar00547)	PZ470735		
	W433 (SYNU-Ar00548)	PZ470736		
	W434 (SYNU-Ar00549)	PZ470737		
*P. taibaiensis*	W224	PP082955	China, Shaanxi, Baoji	Yang et al. [18]
	W225	PP082956		
	W226	PP082957		
	W227	PP082958		
	W228	PP082959		
*P. yanan* sp. nov.	W427 (SYNU-Ar00555)	PZ470730	China, Shaanxi, Yan’an	This study
	W428 (SYNU-Ar00556)	PZ470731		
	W429 (SYNU-Ar00557)	PZ470732		
	W430 (SYNU-Ar00558)	PZ470733		
*P. paralinzhou*	y046	PV031102	China, Henan, Jiaozuo	Yao and Li [7]
*P. taishan*	y133	PV031160	China, Shandong, Taian	Yao and Li [7]

**Table 2:** Average uncorrected p-distances (below diagonal) and K2P distances (above diagonal) among the species discussed in this study. The maximum p-distances within each species are shown in bold on the diagonal.

	*ankang*	*baoji*	*ovatus*	*taibaiensis*	*yanan*	*songi*	*chengkou*
***P. ankang***	**0.001**	0.099	0.102	0.119	0.093	0.101	0.097
***P. baoji***	0.092	**0**	0.096	0.102	0.109	0.090	0.046
***P. ovatus***	0.094	0.090	**0.001**	0.072	0.066	0.105	0.098
***P. taibaiensis***	0.109	0.095	0.068	**0.001**	0.062	0.097	0.100
***P. yanan* sp. nov.**	0.087	0.101	0.062	0.059	**0**	0.109	0.093
***P. songi***	0.093	0.084	0.097	0.090	0.101	**0.009**	0.086
***P. chengkou* sp. nov.**	0.090	0.044	0.091	0.093	0.087	0.080	**0**

## Data Availability

Publication: LSID: urn:lsid:zoobank.org:pub:B1C9E668-9BD0-4B92-B96D-F57FA4261B3F; *Pholcus chengkou* sp. nov., LSID: urn:lsid:zoobank.org:act:ADAB463F-4FEE-41CA-904F-DE4AC5C4BF5A; *Pholcus yanan* sp. nov., LSID: urn:lsid:zoobank.org:act:D01686BC-1DC8-4B07-91C9-DB236D2B98F9. All data produced are available in this manuscript. The sequences are deposited in the GenBank under accession Nos. PZ470730–470740.
